# Phytochemical-Mediated Ah Receptor Activity Is Dependent on Dietary Context

**DOI:** 10.3390/nu17050876

**Published:** 2025-02-28

**Authors:** Fangcong Dong, Andrew J. Annalora, Iain A. Murray, Debopriya Chakraborty, Denise M. Coslo, Craig Marcus, Andrew D. Patterson, Gary H. Perdew

**Affiliations:** 1 Department of Veterinary and Biomedical Sciences, Center for Molecular Toxicology and Carcinogenesis, The Pennsylvania State University, University Park, PA 16802, USA; dong@molbio.mgh.harvard.edu (F.D.);; 2 Department of Environmental and Molecular Toxicology, Oregon State University, Corvallis, OR 97331, USA; andrew.annalora@oregonstate.edu (A.J.A.); craig.marcus@oregonstate.edu (C.M.)

**Keywords:** Ah receptor, ICZ, broccoli, parsley, CYP1A1, apigenin

## Abstract

**Background/Objective:** The aryl hydrocarbon receptor (AHR) is an important mediator of intestinal homeostasis. The AHR senses certain classes of phytochemicals, including many flavonoids and tryptophan metabolites generated in the intestinal tract. Several in vitro studies demonstrate the presence of AHR ligands in numerous plants commonly consumed by humans. However, it has not been established that these foods can activate the AHR in vivo. The aim of this study was to evaluate how phytochemicals in foods can lead to AHR activation in vivo through modulating CYP1A1 activity. **Methods:** Freeze-dried spinach, corn, red potatoes, kidney beans, parsley, onion, carrots, bell peppers, and broccoli were fed to C57BL6/J female mice at 15% *w*/*w* in a semi-purified diet to evaluate the AHR activation potential. In vitro CYP1A1 microsomal assays were utilized to establish specific phytochemicals as CYP1A1 substrates. **Results:** Broccoli, onion, and carrots increased expression of the AHR target gene *Cyp1a1* in the duodenum. Broccoli consumption led to the formation of the potent AHR ligand indolo[3,2-b]carbazole (ICZ), which is also a CYP1A1 substrate. Relative to the other vegetables, parsley contained a high concentration of apiin, a diglycoside of the flavone apigenin. Mice were fed a diet with either 10% parsley, 10% broccoli, or both vegetables. Parsley consumption increased broccoli-mediated *Cyp1a1* induction in the duodenum, liver, and lung. Apigenin is a CYP1A1 substrate that can attenuate ICZ metabolism in vitro and increase broccoli-mediated *Cyp1a1* expression in the lung. **Conclusions:** These results suggest that phytochemical competition for intestinal AHR binding and CYP1A1 metabolism modulates systemic AHR activity.

## 1. Introduction

The aryl hydrocarbon receptor (AHR) is the only ligand-activated member of the basic region helix-loop-helix-PER/ARNT/SIM transcription factor family. This receptor was first identified as the high-affinity receptor for 2,3,7,8-tetrachlorodibenzo-*p*-dioxin (TCDD) and later was demonstrated to be responsible for TCDD-mediated toxicity [1]. More recent studies have revealed the physiologic role of this receptor in a variety of tissues in a context-specific manner. Perhaps the tissue that has received the most attention is the gastrointestinal tract, where AHR activation modulates immune homeostasis, xenobiotic metabolism, and barrier function. In both intestinal epithelial cells and intraepithelial lymphocytes, the AHR can influence cellular differentiation [2,3]. *Ahr^−/−^* mice are more susceptible to intestinal challenge and *Ahr^+/+^* mice treated with the AHR agonist exhibited reduced susceptibility to infection [4]. Furthermore, the targeted deletion of the AHR from intestinal epithelial cells also led to reduced resistance to infection in the colon [2]. Thus, there is considerable interest in sources of AHR activators that can help maintain intestinal homeostasis.

There are four major sources of AHR ligands, endogenous (e.g., kynurenine), microbial (e.g., indole-3-aldehyde), environmental contaminants (e.g., dioxin, polycyclic aromatic hydrocarbons) and dietary (e.g., indole-3-carbinol). The major source of AHR ligand exposure is likely dependent on the environment that a person lives in, their microbiome, and/or their diet. Plants commonly consumed by humans contain a wide variety of phytochemicals, many of which exhibit AHR agonist or antagonist activity in vitro. Perhaps the most abundant class of phytochemicals capable of binding to the AHR are certain flavonoids, such as quercetin, which have been shown to exhibit either weak agonist or antagonist activity [5,6]. Cruciferous vegetables are a rich source of indologlucosinolates that are metabolized by myrosinases upon the disruption of the tissue structure resulting in the release of several metabolites, such as thiocyanates and indole-3-carbinol. Indole-3-carbinol in the acidic environment of the stomach leads to the formation of several condensation products that exhibit a wide range of biological activities [7,8]. Indolo[3,2-b]carbazole (ICZ) is a high-affinity agonist for the AHR, and it is believed to be the primary indole-3-carbinol metabolite that activates the AHR and increases the CYP1A1 levels in the intestinal tract [9,10].

Several studies have indicated that AHR ligands can be found in a variety of plants that are common to our food supply [11,12]. However, these studies utilized plant extracts that were tested in vitro or in cell-based assays. Whether these foods can elicit AHR activation in vivo has not been examined. In our current study, vegetables were added to a semi-purified diet, which was otherwise largely devoid of AHR ligands, as previously reported [13]. The data presented here would suggest that common foods in human diets, such as onions and carrots, can activate the AHR in the intestinal tract, while other foods that have an in vitro activation potential fail to activate the intestinal AHR at the level tested. Another possible mechanism of the phytochemical-mediated increase in AHR activation is through inhibition of CYP1A1 enzymatic hydroxylation of AHR agonists, leading to the increased half-life and thus bioavailability of an AHR agonist. This concept was recently tested by the dietary co-administration of urolithin A, a phytochemical metabolite produced by bacteria in the gut, and broccoli [14]. The presence of urolithin A led to enhanced CYP1A1 activity in tissues outside the intestinal tract, confirming the potential of dietary CYP1A1 substrates to enhance the activation potential of AHR ligands in foods that are also CYP1A1 substrates. In this report, the ability of a flavonoid-rich food to modulate the activity of an AHR-agonist-rich food was tested. Furthermore, whether apigenin, the dominant flavonoid found in parsley and a competitive substrate for CYP1A1, was able to diminish CYP1A1-mediated ICZ hydroxylation was examined. Thus, in principle, the consumption of apigenin-rich parsley along with a source of an AHR agonist would lead to an increase in *Cyp1a1* expression within the intestinal tract or other tissues. Indeed, combined consumption of parsley and broccoli was able to significantly induce *Cyp1a1* in the liver and lung, while a 10% broccoli diet alone did not significantly change *Cyp1a1* in those tissues.

## 2. Materials and Methods

### 2.1. Materials and Reagents

TCDD was provided as a generous gift by Dr. Stephen Safe (Texas A&M University). Apigenin, apiin, α-naphthoflavone (α-NF), kaempferol, isorhamnetin, luteolin and quercetin were obtained from INDOFINE Chemical Company, Inc. (Hillsborough, NJ, USA). Indolo[3,2-b]carbazole was purchased from Carbosynth Ltd. (Berkshire, UK). Apigenin-d5 was purchased from Toronto Research Chemicals (North York, ON, Canada). 5H,11H-indolo[3,2-b]carbazole-2,8-diol (dihydroxyICZ) was synthesized as previously described [14]. The P450-Glo CYP1A1 assay kit, NADPH regeneration system, and luciferase assay were purchased from Promega (Promega, Madison, WI, USA). The cell viability assay was conducted using the CellTiter 96^®®^ Non-Radioactive Cell Proliferation Assay (Promega, Madison, WI, USA). The LC/MS-grade solvents, including methanol and acetonitrile, were purchased from Fisher Scientific (Hampton, NH, USA). The standard solutions of apigenin for the liquid chromatography were gradually diluted with 3% methanol (*v*/*v*) containing 1 μM apigenin-d5 to generate the calibration curve.

### 2.2. Mice

C57BL6/J mice were originally purchased from Jackson Laboratory (Bar Harbor, ME, USA) and were bred in-house. The mice were maintained in autoclaved polypropylene cages with corncob bedding and a paper tube enrichment in a pathogen-free environment along with ad libitum access to diet and water.

### 2.3. Diets and Feeding Studies

The semi-purified AIN-93G diet was obtained from Dyets Inc. (Bethlehem, PA, USA) and was ground into a powder with a Ninja Professional blender. The broccoli cultivar Imperial, a kind gift from John Esslinger and Brian Campbell, was freshly harvested from the field then freeze-dried and ground to a powder. Dried kidney beans were hydrated overnight, then boiled for 15 min prior to freeze-drying. Fresh corn was cut from the cob, while spinach, carrots, red potatoes, yellow onion, bell pepper and curly leaf parsley obtained from a market were all minced and freeze-dried. All the vegetables were then ground into a powder prior to mixing into a ground semi-purified diet. The animal experiments were performed using protocols approved by the Pennsylvania State University Institutional Animal Care and Use Committee under protocol number 201800236. Forty-nine 8-week-old C57BL/6J female mice were placed in a large cage and randomized into groups of five or six mice based on power analyses and then administered an AIN-93G diet for seven days. The mice were then placed on a diet containing 15% (*w*/*w*) onion, carrot, kidney bean, bell pepper, potato, spinach, corn, or broccoli, or they remained on a control diet for 3 days. The mice in each treatment group were co-housed 3 per cage with controlled temperature (24 °C) and lighting (12-h light dark cycle) and 70 air change/h. The mice per group was determined by power analysis of previous studies that utilized *Cyp1a1* mRNA analysis. Duodenum and colon scrape samples and other tissues were collected from individual mice and immediately frozen in liquid nitrogen after sacrifice, followed by RNA extractions. For the mouse parsley diet study, a total of thirty-six 8-week-old C57BL/6 female mice were randomized into groups of four to five mice per cage and acclimatized to a purified diet for seven days and then administered finely ground diets containing concentrations of 10% (*w*/*w*) freeze-dried broccoli, 10% (*w*/*w*) curly parsley and 10% (*w*/*w*) mixtures of broccoli and curly parsley in an AIN-93G diet for an additional seven days (n = nine per experimental group). The animals were euthanized by carbon dioxide asphyxiation, then samples of liver, lung, kidney, cecal contents, and duodenum and colon scrapes were collected and immediately snap-frozen in liquid nitrogen. Blood was also collected in BD microtainer SST^TM^ tubes, and serum was obtained by centrifugation at 4000× *g* for 10 min. The supernatant was then transferred into microcentrifuge tubes and frozen in liquid nitrogen. All the biological samples were stored in a freezer at −80 °C until further analyses. In all the mouse studies, no data points were excluded from the analysis. Also, in all the mouse experiments, Denise Coslo maintained the mouse colony and set up the mice randomized into cages and the appropriate diets. The tissues were harvested by Iain Murray and Fangcong Dong. Fangcong Dong performed the qRT-PCR analysis.

### 2.4. Cell Culture and Luciferase AHR Reporter Assays

Hepa 1.1 cells were generated and cultured as previously described and obtained from Dr. Michael S. Denison (UC, Davis) [15]. Caco2 cells were obtained from the American Type Culture Collection. The stable reporter cell line mouse Hepa 1.1, as well as the human epithelial colorectal adenocarcinoma cell line Caco2, was maintained in α-minimal essential medium (Sigma-Aldrich, St. Louis, MO, USA), supplemented with 10% fetal bovine serum (HyClone Laboratories, Logan, UT, USA), 100 U/mL penicillin, and 100 µg/mL streptomycin (Sigma, St. Louis, MO, USA). The AHR reporter cell lines used in the luciferase reporter assays were seeded in twelve-well plates and treated the following day with TCDD, apigenin and apiin dissolved in dimethyl sulfoxide (DMSO) (0.1% final concentration in cell culture) and incubated for 4 h. The luciferase activity was measured in the lysate after freeze–thaw using a TD-20e luminometer and luciferase assay substrate (Promega, Madison, WI, USA) according to the manufacturer’s instructions.

### 2.5. RNA Isolation and Quantitative PCR

For the RNA isolation, the samples were placed in TRI Reagent (Sigma, MO, USA) and homogenized for 30 s at 6500 rpm using 10–15 1 mm silica/zirconia beads (Biospec Products, OK, USA) with a BeadBlaster-24 homogenizer (Benchmark, NJ, USA). The resulting homogenates were then re-purified using RNeasy columns (Qiagen, Hilden, Germany) according to the manufacturer’s instructions. The purified total RNA was used to generate cDNA. Quantitative real-time PCR was performed with primers as described previously (Hubbard et al., 2017 [13]), normalized to *β-actin* or *Rpl13a*. The PCR was conducted using Perfecta SYBR Green (QuantaBio, MD, USA).

### 2.6. Isolation of Microsomes

Caco2 cells and Hepa1 cells were seeded in a concentration of 10^5^ cells/cm^2^ and treated with 5 nM TCDD for 24 h to induce CYP1A1 activity. The microsomes were isolated as follows: the cells were washed with phosphate-buffered saline, trypsinized and pelleted by centrifugation at 900× *g* for 3 min. The pellet was re-washed with phosphate-buffered saline, re-pelleted, then resuspended in 0.25 M sucrose and 10 mM Tris-HCl (pH 7.5), with protease inhibitors. The cells were manually homogenized on ice. The suspension was centrifuged at 10,000× *g* for 10 min at 4 °C and the supernatant was then centrifuged at 162,000× *g* for 90 min at 4 °C. The resulting microsomal pellet was resuspended in the above buffer and the protein concentration was assessed. Aliquots of the microsomes were stored at –80 °C for the CYP1A1/1B1 activity assay.

### 2.7. CYP1A1 Activity Assay

First, 12.5 μL of CYP reaction mixture, including the microsome, Luc-CEE and KPO_4_ buffer, and 12.5 μL of test compound were combined and pre-incubated at 37 °C for 10 min in a white 96-well plate. The reactions were initiated by adding an equal volume of NADPH regeneration system (25 μL) and placed at 37 °C for 25 min. A final reaction volume of 50 µL was used for reactions containing 10 μg of isolated microsomes, 100 mM KPO_4_ (pH 7.4) buffer, 30 μM Luc-CEE, test compound, and NADPH regeneration system, which were performed in triplicate. The reactions were terminated by the addition of 50 μL of luciferin detection reagent and incubated at 37 °C for 10 min. The luminescence was recorded using a luminometer, and a minus-P450 control without microsome was used to measure the background. The relative CYP1A1 activity was expressed as the percentage of the luminescence after incubation with vehicle.

### 2.8. Apigenin Competitive Metabolism Assay

First, 12.5 μL of ICZ or a mixture of ICZ and apigenin was combined with 12.5 μL of CYP reaction mixture without Luc-CEE and pre-incubated at 37 °C for 10 min. The reactions were initiated by adding 25 μL of NADPH regeneration system and placed at 37 °C for 120 min of incubation. The incubations were quenched by adding 800 μL of ice-cold 100% methanol (*v*/*v*) containing 1 μM chlorpropamide as the internal standard. The mixture was then vortexed for 15 s and placed at −20 °C for 30 min, followed by centrifugation at 12,000× *g* for 20 min at 4 °C. The supernatants were dried using a SpeedVac and then resuspended in 60 µL of 50% methanol (*v*/*v*) for the LC/MS/MS analysis.

### 2.9. Apigenin Quantification Using LC/MS/MS

Approximately 50 mg of fresh cecal contents was extracted twice with 500 µL of ice-cold 80% (*v*/*v*) methanol containing 1 µM apigenin-d5, homogenized with 1 mm zirconium beads using a BeadBlaster^TM^ 24 (Benchmark Scientific, Edison, NJ, USA) homogenizer, followed by centrifugation at 17,000× *g* for 10 min at 4 °C. The supernatants were collected and combined. For the serum samples, 20 µL of serum was mixed with 80 µL chilled methanol containing 1 µM apigenin-d5. The mixture was then vortexed for 15 s and stored at −20 °C for 30 min. The samples were then centrifuged at 17,000× *g* for 10 min at 4 °C. The supernatants from the cecal contents and serums were dried using a SpeedVac and then resuspended in 100 µL of 3% methanol for apigenin quantitation by LC/MS. Next, 5 µL of supernatant was injected into reverse-phase UHPLC using a Prominence 20 UFLCXR system (Shimadzu, Columbia, MD, USA) with a Waters (Milford, MA, USA) BEH C18 column (2.1 × 100 mm × 1.7 µm particle size) maintained at 55 °C, with a 20 min aqueous acetonitrile gradient, at a flow rate of 250 µL/min. Solvent A was HPLC-grade water with 0.1% formic acid and solvent B was HPLC-grade acetonitrile with 0.1% formic acid. The initial conditions were 97% A and 3% B, increasing to 45% B at 10 min, 75% B at 12 min, where it was held at 75% B until 17.5 min before returning to the initial conditions. The eluate was delivered into a 5600 (QTOF) TripleTOF using a Duospray™ ion source (SCIEX, Framingham, MA, USA). The capillary voltage was set at 5.5 kV in positive ion mode with a declustering potential of 80 V. The mass spectrometer was operated with a 100 ms TOF scan from 50 to 500 *m*/*z* and 16 MS/MS product ion scans (100 ms) per duty cycle using a collision energy of 50 V with a 20 V spread.

### 2.10. Computational Docking Analysis

The ligand binding affinity and target oxidation sites for apigenin were investigated using Autodock 4.2 and Autodock Vina for both the human and mouse models of CYP1A1 and CYP1B1 [16,17,18]. The crystal structures of CYP1A1 (4I8V) and CYP1B1 (3PM0) were used to prepare the docking models for human enzymes and as templates for the mouse homology models using the SWISS-MODEL server [19,20,21]. The homology models were validated for the overall quality, clashes and outliers using the SAVESv6.0 server (https://saves.mbi.ucla.edu/, accessed on 19 March 2020) and ChimeraX using the ISOLDE plugin [22,23,24,25]. The computational models of apigenin and luteolin were obtained from the PubChem database [26]. Autodock 4.2 was run using the standard settings, using the Genetic Algorithm search parameter (long evaluations, 20–100 GA runs) as previously described [27]. The grid box parameters were generated in Autogrid 4 for both Autodock 4.2 and Autodock Vina, using a 60–80 Å^3^ grid centered on the heme center. The docking results were analyzed using Autodock 4.2, the PyMOL Molecular Graphics System, Version 2.52 Schrödinger, LLC (New York, NY, USA) [28].

### 2.11. Statistical Analysis

Data were compared using either one-way analysis of variance with Tukey’s multiple comparison post-test or Student’s *t* test in GraphPad Prism 6.01 (GraphPad Software, Inc., La Jolla, CA, USA) to determine statistical significance between different groups. The value of *p* < 0.05 was used as the minimal level of significance. The power analysis/sample size analysis was performed (https://homepage.univie.ac.at/robin.ristl/samplesize.php?test=ttest, accessed on 10 June 2020) using *Cyp1a1* data previously published with the following parameters: *Alpha* value of 0.05, standard deviation of 3, which results in a minimum sample size, n = 2 [13].

## 3. Results

### 3.1. Plant Foods as a Source of AHR Ligands

**Figure 1:**
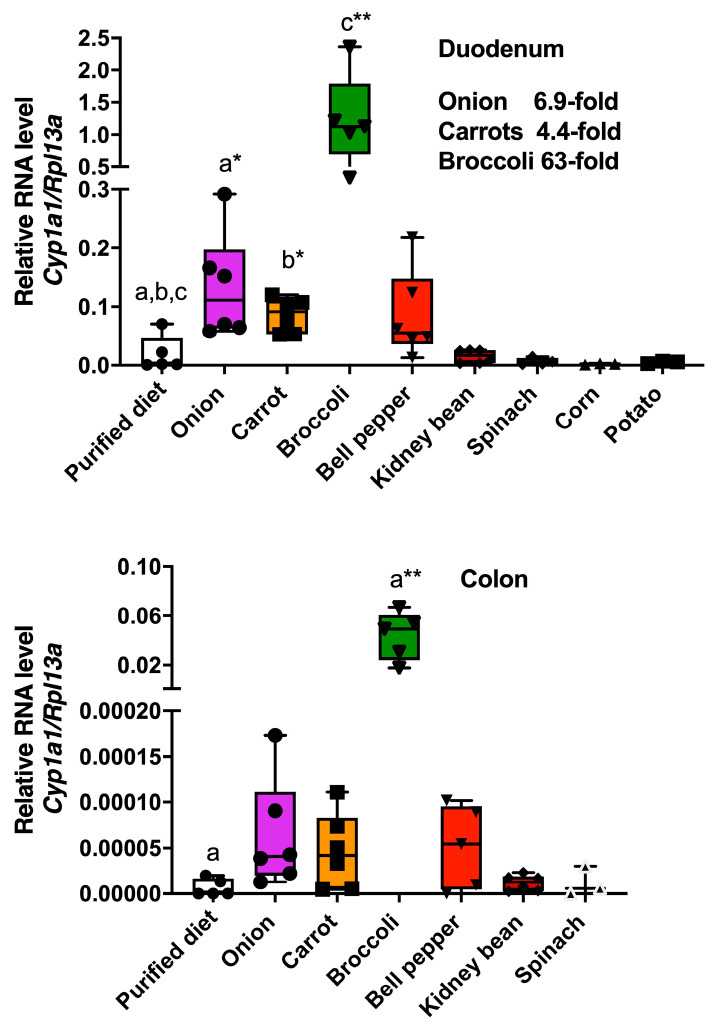
Some common foods are capable of increasing AHR activation in the intestinal tract. Foods as indicated were added at 15% on a dry weight basis to a semi-purified diet and fed to mice for 3 days. Duodenum and colonic tissues were isolated and RNA extracted, followed by qRT-PCR determination of the *Cyp1a1* mRNA levels. The data are the mean ± SEM, one-way ANOVA, Tukey’s test, * *p* < 0.05, ** *p* < 0.01. Alphabetical characters indicate statistical comparisons between two groups.

The vegetables were incorporated at 15% of a semi-purified diet on a *w*/*w* basis. This level of exposure was chosen because 15% broccoli in the diet was previously shown to significantly activate the AHR in the intestinal tract [13]. In this study, the Imperial broccoli cultivar was used and contained 9.9 µmol/g glucobrassicin on a dry weight basis, similar to the level present in our previous studies utilizing the Lieutenant cultivar [13]. It is important to note that the effects of freeze-drying on the phytochemical availability and stability are not known. The mice were placed on an ad libitum semi-purified diet for one week prior to being fed a 15% broccoli, spinach, corn, red potato, kidney bean, onion, carrot or bell pepper diet for 3 days. Duodenal and colonic tissues were taken and the expression level of the AHR target gene *Cyp1a1* was assessed (Figure 1). Both onion and carrot consumption significantly increased the *Cyp1a1* mRNA levels relative to a semi-purified diet, while broccoli induced a 63-fold increase in *Cyp1a1* expression. In contrast, bell peppers, kidney beans, corn, spinach, and red potato failed to mediate a statistically significant level of *Cyp1a1* expression. In the colon only, broccoli was capable of significantly increasing *Cyp1a1* expression. There are two possible explanations for the modest duodenal AHR activation by onions and carrots. First, there are agonists present, and second, there are CYP1A1 substrates that decrease the metabolism of a potent AHR ligand.

### 3.2. Abundant Flavonoids Do Not Exbibit AHR Agonist Activity at Relevant Concentrations

**Figure 2:**
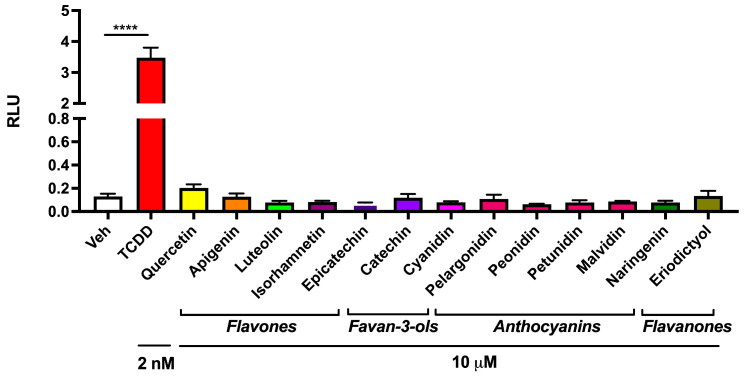
Assessment of the flavonoid-mediated agonist activity in an AHR-driven reporter cell line. The ability of 13 flavonoids at 10 µM to increase AHR activity was assessed in the Hepa 1.1 AHR reporter cell line. Cells were treated for 4 h with each flavonoid, compared to 2 nM TCDD as a positive control, and the luciferase activity was determined. The data are the mean ± SEM, one-way ANOVA, Tukey’s test, **** *p* < 0.0001.

Several flavonoids can modulate AHR activity and are abundantly present in vegetables. A survey published by the USDA lists the levels of the major flavonoids found in a variety of plants consumed by humans [29]. This database shows that some of the most abundant flavonoids, which are most likely to exhibit AHR-modulating activity, are found in vegetables. The ability of these flavonoids to activate the mouse AHR in the Hepa 1.1 DRE-driven reporter cell line was examined, and none of the flavonoids at 10 µM exhibited agonist activity (Figure 2). Next, the ability of each flavonoid to suppress TCDD-mediated induction of luciferase activity in mouse Hepa 1.1 reporter cells was examined and only the flavones tested were capable of inhibiting AHR-mediated activity (Figure S1). As a control experiment, flavonoids were tested for the ability to directly inhibit luciferase enzymatic activity, and no inhibition was observed (Figure S2). The ability of this set of flavonoids to modulate AHR transcriptional activity in human HepG2 40/6 DRE-driven reporter cells revealed that the flavones exhibited no significant agonist activity at a 10 μM concentration, except quercetin, which mediated modest activity (Figure S3). These results indicate that the flavonoids tested may potentially attenuate AHR activation in the presence of a potent ligand.

### 3.3. Abundant Flavonoids Are Inhibitors of or Substrates for CYP1A1

**Figure 3:**
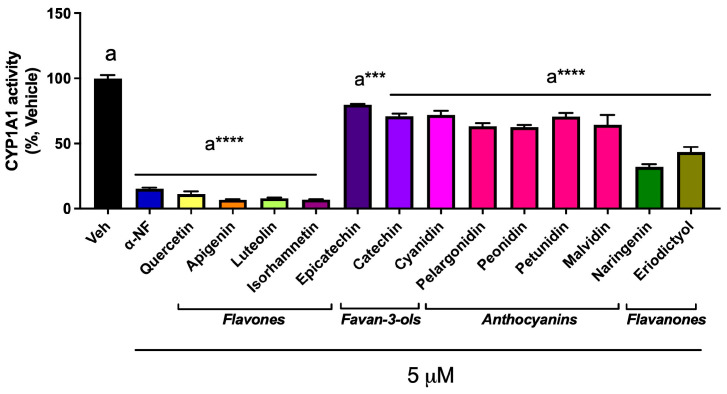
Flavonoids differentially inhibit CEE-luciferin metabolism in a Hepa 1 microsomal assay system. Thirteen abundant flavonoids were assessed for the ability to inhibit CEE-luciferin metabolism in an in vitro microsomal assay system. The data are the mean ± SEM, one-way ANOVA, Tukey’s test, *** *p* < 0.001, **** *p* < 0.0001. Alphabetical characters indicate statistical comparisons between two groups.

Another possible mechanism of AHR activation by food consumption is through the ability of certain flavonoids to inhibit or compete for CYP1A1 enzymatic activity in the intestinal tract and diminish the metabolism of an AHR agonist. To examine this mechanism, the CYP1A1/1B1 metabolism assay system utilized the Promega CEE-luciferin substrate along with microsomes from mouse Hepa 1 cells expressing CYP1A1/1B1. CEE-luciferin is a highly specific substrate for CYP1A1/1B1 metabolism. The flavones, quercetin, apigenin, kaempferol, luteolin, and isorhamnetin, exhibited marked inhibition of mouse CYP1A1/1B1 metabolism of CEE-luciferin at 5 µM, and α-naphthoflavone (αNF) was utilized as a positive control in this assay (Figure 3). In the dose–response assays, these flavones inhibited mouse CYP1A1/1B1 with an IC_50_ of between 2 and 5 µM, respectively (Figure S4). In contrast, the other classes of flavonoids exhibited modest inhibitor potential. Thus, these data indicate that flavones are relatively potent substrates for or inhibitors of CYP1A1/1B1.

### 3.4. Parsley/Broccoli Diet Induces Cyp1a1 Expression

**Figure 4:**
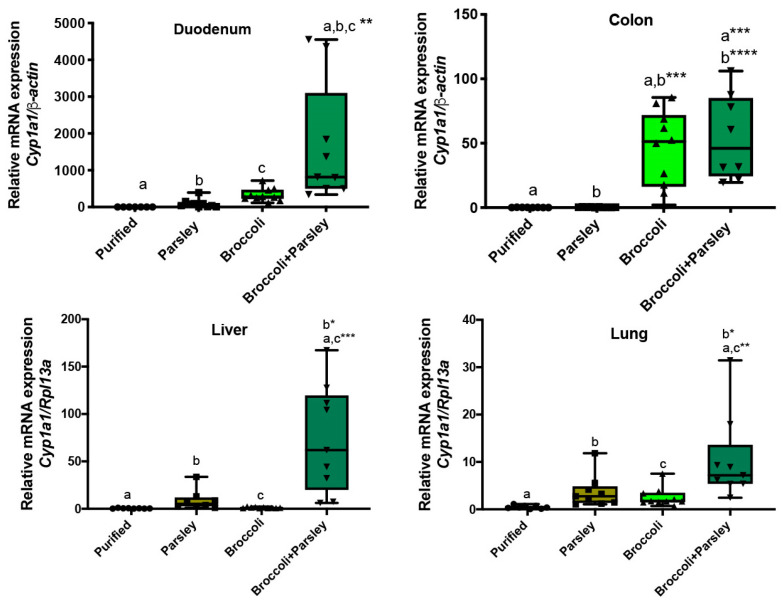
Parsley enhances broccoli-mediated *Cyp1a1* expression in the duodenum, liver and lung. Parsley, broccoli, or parsley + broccoli at 10% each in a semi-purified diet were fed for 7 days. RNA was isolated from the duodenum, colon, liver, and lung; qRT-PCR assessed the level of *Cyp1a1* expression. The data are the mean ± SEM, one-way ANOVA, Tukey’s test, * *p* < 0.05, ** *p* < 0.01, *** *p* < 0.001, **** *p* < 0.0001. Alphabetical characters indicate statistical comparisons between two groups.

Apigenin is an abundant flavonoid that exists in many plants as the diglycoside apiin. Apigenin is the major flavonoid in parsley, found at between 4.5 and 13% on a dry weight basis, and thus represents a major source of essentially a single flavonoid and therefore is an ideal food source to test the hypothesis that a dietary flavone can modulate AHR activity through CYP1A1 substrate competition in a dietary context [29]. Thus, we examined whether parsley would modulate the broccoli-mediated AHR activation potential. The mice were fed either broccoli at 10%, parsley at 10%, or a combination of the two in a semi-purified diet, which was otherwise devoid of AHR ligand activity. This level of exposure was chosen because an increase in hepatic *Cyp1a1* expression was not observed in a previous study utilizing a diet with 10% broccoli [14]. The level of *Cyp1a1* in the duodenum after feeding a parsley or broccoli diet identified a marginal but not statistically significant difference compared to the control diet (Figure 4). In contrast, the combined broccoli/parsley diet induced *Cyp1a1* expression 1700-, 18- and 5-fold when compared to the purified, parsley and broccoli diets, respectively. A comparison between the parsley and broccoli/parsley diets resulted in a 164-fold increase in *Cyp1a1* expression in the colon. However, a similar increase in *Cyp1a1* expression was observed in the colon with both the broccoli and broccoli/parsley diets. A comparison between parsley versus broccoli/parsley in the liver and lung led to a 61- and 4-fold increase in *Cyp1a1*, respectively. It is worth noting that the response to the parsley/broccoli diet was quite variable from mouse to mouse, although the reason for this is unknown at this time. Although not statistically significant, the 10% parsley diet did show a trend toward an increase in *Cyp1a1* expression in the lung. Interestingly, all the diets exhibited a similar level of *Cyp1a1* expression in the kidney (Figure S5).

### 3.5. Parsley and the Generation of Apigenin in the Intestinal Tract

**Figure 5:**
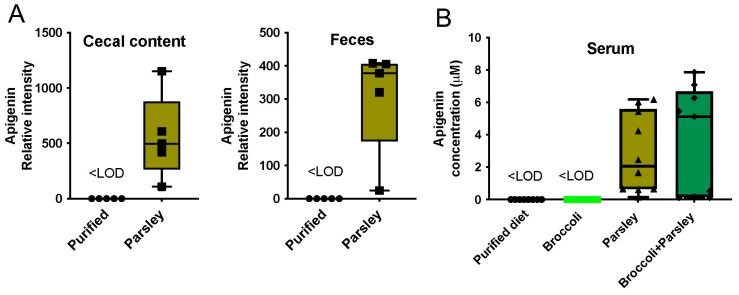
Presence of apigenin in the intestinal tract and in the serum after parsley consumption. LC/MS/MS analysis revealed that a 10% parsley/semi-purified diet led to the presence of apigenin in cecal and fecal matter (**A**). A 10% parsley or a 10% broccoli + 10% parsley diet resulted in apigenin serum levels in a low µM range (**B**). Apigenin was less than the limit of detection (<LOD) in a purified and broccoli diet.

Since apigenin exists in parsley predominantly as the diglycoside apiin, we tested whether apigenin can be detected in mice fed a parsley diet. Indeed, apigenin was detected in both the cecal and fecal extracts in mice fed a parsley diet (Figure 5A). The level of apigenin absorbed in mice on a 10% parsley or 10% parsley and broccoli diet was also examined in the serum, and the level detected varied considerably with an average of ~2 or 5 µM, respectively (Figure 5B). While the reason for the wide variation in the serum levels is not known, it may be due to the timing of food consumption between mice.

### 3.6. Apigenin Is a Substrate for or Competitive Inhibitor of CYP1A1/1B1

**Figure 6:**
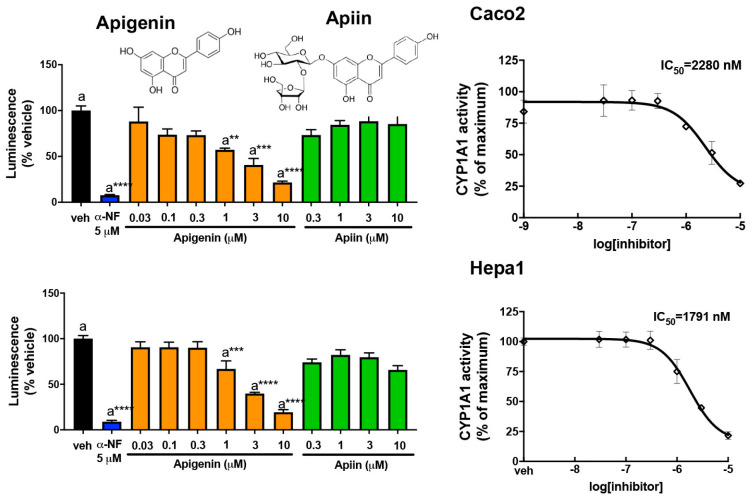
Apigenin, but not apiin, inhibits CEE-luciferin metabolism in a microsomal assay system. Increasing concentrations of apigenin or apiin were added to either the human (Caco2) or mouse (Hepa1) in vitro microsomal assay system and the luciferase activity was measured. α-NF was utilized as a positive control. The data are the mean ± SEM, one-way ANOVA, Tukey’s test, ** *p* < 0.01, *** *p* < 0.001, **** *p* < 0.0001. Alphabetical characters indicate statistical comparisons between two groups.

Apigenin inhibits CYP1A1 in both human and mouse CYP1A1/1B1 microsomal assays with an IC_50_ of ~2 µM (Figure 6), while apiin shows no inhibition. A direct comparison between species cannot be made since the level of CYP1A1 protein in the microsomes is not the same as was previously published [14].

### 3.7. Molecular Modeling of CYP1A1/1B1 Metabolism of Apigenin

**Figure 7:**
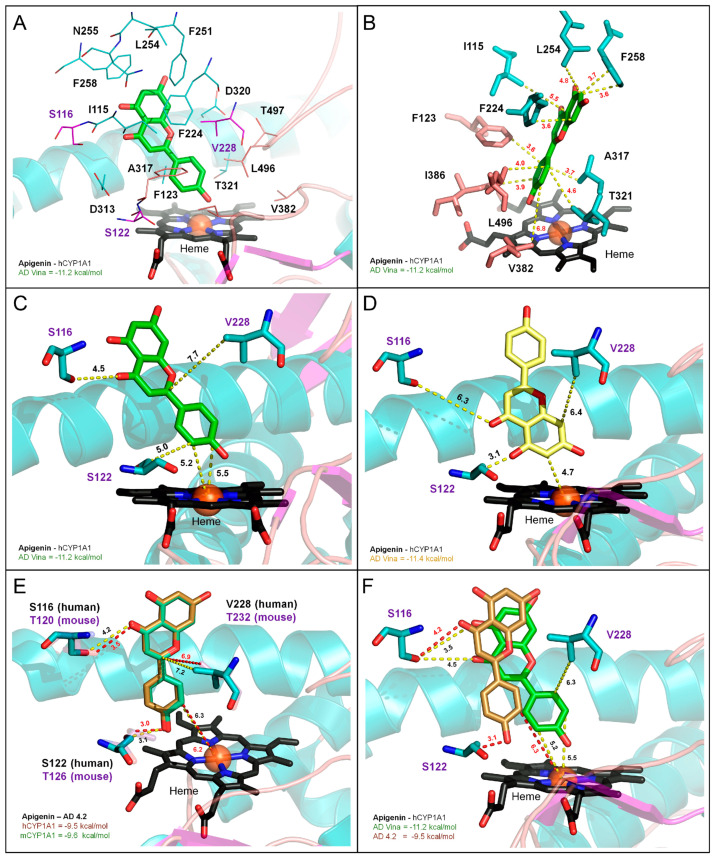
Apigenin docking in human and mouse CYP1A1. The substrate binding properties of apigenin were explored using Autodock (versions 4.2 and Vina) and molecular models of human CYP1A1 (hCYP1A1) and mouse CYP1A1 (mCYP1A1), which were created using the crystal structure of CYP1A1 (1TQN; see Section 2). Apigenin docks to the CYP1A1 active site with low nanomolar affinity in both human (−11.2 kcal/mol; 6.3 nM; green stick) and mouse (−11.1 kcal/mol; or 7.5 nM; Table 1) forms of the enzyme (see Table 1). Here, the most common binding pose for apigenin is shown (−11.2 kcal/mol; green stick) in the CYP1A1 active site formed by amino acids I115, S116, S122, F123, F224, V228, F251, L254, N255, F258, D313, A317, D320, T321, V382, L496, and T497. The species-specific amino acid differences between the human and mouse CYP1A1 active site are limited to 3 residues (S116, S122 and V228), which are highlighted (purple lines) (**A**). Apigenin interacts with a cluster of highly conserved, hydrophobic residues (I115, F123, F224, V228, F251, L254, F258, A317, V382, L496) that define the narrow, active site pocket of both hCYP1A1 and mCYP1A1 (**B**). For the most common binding configuration (−11.2 kcal/mol; green stick), apigenin’s B-ring is positioned over the heme center, with the C3 and C5 target carbons in proximity (5.2–5.5 Å, respectively) for oxidation (**C**). A lower energy, but less common, docking solution (−11.4 kcal/mol; yellow stick) was also obtained for apigenin in the human model, but not in the mouse model. In this low-energy solution, the A-ring of apigenin is positioned over the heme, placing the C6 carbon within 4.7 Å of the heme center (**D**). Despite this outlier, apigenin docking was generally consistent for both mCYP1A1 and hCYP1A1, using either version of Autodock. Minor differences among the interactions between the substrate and the polymorphic active site residues S116(T), V228(T), and S122(T) were detected but did not significantly alter the terminal substrate positioning. Here, the nearly identical Autodock 4.2 results for mouse and human CYP1A1 are shown (hCYP1A1: 107 nM; −9.5 kcal/mol (orange stick); and mCYP1A1: 90 nM; −9.6 kcal/mol (light green stick), with the C3 target carbon of apigenin positioned 6.2–6.3 Å from the heme center, respectively) (**E**). Autodock Vina predicted lower energy binding of apigenin to hCYP1A1 than Autodock 4.2 (−11.2 kcal/mol (green stick) vs. −9.5 kcal/mol (orange stick), respectively), but both programs consistently positioned the C3 or C5 target carbons of the B-ring ~5.2–6.3 Å from the heme center (**F**).

To establish that apigenin is a CYP1A1/1B1 substrate and gain insight into the likely metabolites that would be formed, a computational docking approach was applied. The substrate binding properties of apigenin were studied with Autodock 4.2 and Autodock Vina. Structure-based docking models of the human cytochromes P450 1A1 (hCYP1A1) and 1B1 (hCYP1B1), and homology models of the mouse cytochromes P450 1A1 (mCYP1A1) and 1B1 (mCYP1B1), were developed to explore the potential differences in human and mouse substrate recognition and metabolism. Using Autodock Vina, we calculated that apigenin binds mouse and human CYP1A1 with low nanomolar affinity (hCYP1A1: 6.3 nM; −11.2 kcal/mol; mCYP1A1: 7.5 nM; −11.1 kcal/mol; see Table 1) in a common docking conformation just above the heme center (Figure 7A,B).

In this configuration, the B-ring of apigenin is positioned over the heme, with the target carbons C3 and C5, 5.2 to 5.5 Å from the iron center, respectively (Figure 7C). A second, higher affinity but less common, binding event was also obtained for apigenin in the hCYP1A1 model only (hCYP1A1: 4.5 nM; −11.4 kcal/mol). Apigenin can also dock with the A-ring positioned over the heme, with the putative target carbon C6 positioned just 4.7 Å away from the catalytic center (Figure 7D). This docking event was not recapitulated in the mCYP1A1 model, suggesting polymorphic residues in the mouse active site (T120, T126, and T232) may modulate the substrate recognition and the regiospecificity of the target oxidation. This subtle, species-specific effect was not seen when using the related docking program, Autodock 4.2, which found a common, lower-affinity docking solution for apigenin in both mCYP1A1 and hCYP1A1 (hCYP1A1: 107 nM; −9.5 kcal/mol; and mCYP1A1: 90 nM; −9.6 kcal/mol; see Figure 7E). While a significant difference in the substrate binding affinity was noted, both docking programs consistently positioned the C3 and C5 target carbons of apigenin closest to the heme center, despite subtle differences in the interaction with the polymorphic active site residues (S116, S122 and V228; see Figure 7F). These results predict that apigenin metabolism may be slightly more diverse in humans than mice but that both enzymes are well configured to convert apigenin to luteolin through regiospecific oxidation of the flavonoid B-ring.

To further explore the nature of the flavonoid binding in the CYP1 family of enzymes, we also compared the apigenin recognition in computational docking models of mouse and human CYP1B1. As shown in Figure S6A, apigenin docks the CYP1B1 active site with higher affinity than CYP1A1 (hCYP1B1: 2.3 nM; −11.8 kcal/mol; mCYP1B1: 2.7 nM; −9.7 kcal/mol; see Table S1), but in the same general configuration shown in Figure 7A,C. This common pose for apigenin is stabilized by the same group of hydrophobic, active site residues that are highly conserved in all the CYP1 family genes (Figure S6B). The electrostatic contacts between apigenin and hydrophilic CYP1B1 active site residues were also examined (Figure S6C). Most of these residues (S127, S131, N265, and D326) are well conserved in both CYP1A1 and CYP1B1 and likely play a similar role in substrate recognition. The polymorphic residues in the helix F (N228 in mCYP1B1 and hCYP1B1; N221 in hCYP1A1; S225 in mCYP1A1) and helix I (Q332 in mCYP1B1 and hCYP1B1; F319 in hCYP1A1; F323 in mCYP1A1) were also shown to contact apigenin but did not alter the general nature of flavonoid docking seen in the two enzymes. It is notable that apigenin was calculated to have a substrate binding affinity nearly 30-fold higher in the human CYP1B1 model than the mouse model (2.7 nM vs. 77.7 nM, respectively), despite the similar docking solutions that are virtually superimposed (Figure S6D). The differential levels of steric repulsion created by the polymorphic β1–4 sheet residue at position 395, which is V395 in humans and L395 in mice, appear to account for the large calculated difference in substrate binding among the two models. While the presence of polymorphic residue L395 in the mouse CYP1B1 enzyme appears to cause added steric repulsion of the substrate in the proximal surface of the active site, it is not predicted to alter the regiospecificity of apigenin target oxidation among the mice and human isoforms of CYP1A1 or CYP1B1.

### 3.8. Apigenin Is Metabolized by CYP1A1/1B1 to Luteolin

To determine whether apigenin is a competitive substrate or a simple inhibitor in the human CYP1A1/1B1 assay system, we adopted an LC-MS/MS approach to determine if apigenin is hydroxylated in this assay and forms hydroxylated derivatives in the mouse CYP1A1/1B1 assay system. The results indicate that in the context of a functional microsomal CYP1A1/B1 system, apigenin is actively metabolized to the flavonoid luteolin (Figure S7), as confirmed by comparing its retention time and MS/MS spectra with those of an authentic standard.

### 3.9. Apigenin Inhibits ICZ Metabolism

**Figure 8:**
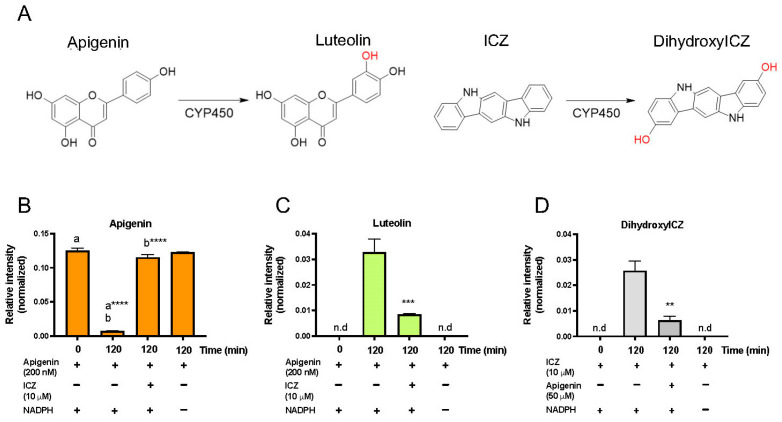
Apigenin can inhibit ICZ metabolism. The Hepa 1 microsomal assay system is capable of metabolizing apigenin to luteolin, and ICZ to dihydroxyICZ (**A**). Apigenin was incubated with the microsomal assay system under the conditions given for 120 min. LC-MS/MS was utilized to assess the level of apigenin (**B**), luteolin (**C**), and dihydroxyICZ (**D**). The data are the mean ± SEM, one-way ANOVA, Tukey’s test, ** *p* < 0.01, *** *p* < 0.001, **** *p* < 0.0001. Alphabetical characters indicate statistical comparisons between two groups.

Upon consumption of broccoli, the high-affinity AHR agonist ICZ is formed in the acidic environment of the stomach, and we have previously demonstrated that ICZ is a substrate for CYP1A1/1B1 that leads to the formation of dihydroxyICZ [14]. Co-incubation of apigenin with ICZ in a microsomal CYP1A1/1B1 assay system revealed that ICZ inhibited apigenin metabolism to luteolin (Figure 8A–C). Conversely, apigenin attenuated the formation of the dihydroxyICZ from ICZ (Figure 8D). Thus, apigenin is an effective competitive substrate capable of attenuating ICZ metabolism.

### 3.10. Apigenin Increases AHR Activation in the Lung

**Figure 9:**
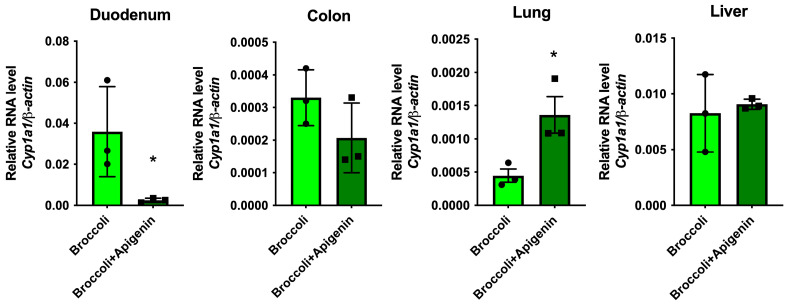
Dietary apigenin is capable of increasing the lung *Cyp1a1* levels in the presence of broccoli. Here, 10% broccoli or 10% broccoli + 5 mg/g of apigenin in a powdered diet was fed to mice for 3 days and the *Cyp1a1* levels were assessed in duodenal scrapes, colonic scrapes, liver and lung. The data are the mean ± SEM, Student’s *t* test, * *p* < 0.05. Alphabetical characters indicate statistical comparisons between two groups.

To confirm that dietary apigenin is capable of increasing *Cyp1a1* expression in tissues outside the intestinal tract, the mice were fed either a 10% broccoli diet or a diet with 10% broccoli + 5 mg apigenin/g (*w*/*w*) of powdered diet. The amount of apigenin added was estimated from the amount of apigenin in a 10% parsley diet, utilizing the USDA flavonoid tables. After three days, RNA was isolated from tissues and quantitative RT-PCR was used to assess the level of *Cyp1a1* expression. Apigenin significantly suppressed broccoli-mediated *Cyp1a1* expression in the duodenum but had no significant effect on colonic or liver *Cyp1a1* expression (Figure 9). In contrast, the presence of apigenin in the diet significantly increased *Cyp1a1* expression in the lung compared to the broccoli diet.

## 4. Discussion

Several recent studies have firmly established the importance of the AHR in intestinal homeostasis and the response in injury. For example, AHR agonists in the gastrointestinal tract appear to enhance barrier function in part through enhanced IL22 expression, which in turn helps protect the gut from pathogenic bacterial infection and genotoxic stress [2,30,31]. In addition, there is evidence that AHR activation in the intestinal tract attenuates DNA damage in the intestinal epithelium, revealing that multiple protective AHR-mediated mechanisms occur in the small intestine [32].

Immune surveillance appears to be improved by the presence of AHR ligands, which thus offer the potential to maintain gut homeostasis [33]. Furthermore, the overexpression of intestinal CYP1A1 in a mouse model leads to the depletion of the AHR activation potential and increases the susceptibility to bacterial invasion [30]. In contrast, the presence of an AHR antagonist in the gut may be of use from a therapeutic standpoint in specific situations, such as cancer treatment. For instance, the use of AHR antagonists exhibits anti-tumor activity for several tumor types and in combination with immunotherapy [34,35,36,37].

A plant-based diet could theoretically be a rich source of AHR ligands and potentially exhibit therapeutic properties. However, one would need to select the appropriate vegetables, such as cruciferous vegetables, to achieve this goal. Other than cruciferous vegetables, there have been a limited number of in vivo feeding studies testing whether specific foods are capable of modulating AHR activity. For example, feeding mice a cacao polyphenol extract resulted in the suppression of 3-methylcholanthrene-mediated induction of *Cyp1a1*, suggesting the presence of an AHR antagonist [38]. Also, a 15% broccoli diet has previously been shown to robustly induce *Cyp1a1* expression in the intestinal tract when compared to a semi-purified diet and is protective against chemical insult [13]. This would suggest that one can obtain a sufficient level of dietary AHR ligands to influence intestinal health. Thus, one focus of this study was to determine whether other commonly consumed vegetables can directly or indirectly activate the AHR in vivo. To test this hypothesis, mice were placed on a semi-purified diet deficient in phytochemicals that can modulate AHR activity. This yielded a very low baseline of *Cyp1a1* expression for testing for the presence of AHR ligands or activators in specific foods that could not be achieved with a standard plant-based rodent chow diet. The foods chosen to be tested in this study needed to meet two criteria. First, they must be vegetables commonly consumed by humans, and second, an extract of these vegetables must have been previously shown to activate the AHR in vitro [11,12]. In contrast to broccoli, spinach, corn, red potatoes, bell peppers, and kidney beans failed to significantly activate the AHR in the intestinal tract, suggesting either the absence of positive AHR modulators or the presence of AHR antagonists. However, onions and carrots elicited a modest level of AHR activation, but significantly lower than that achieved with broccoli. These results underscore that in vivo cruciferous vegetables appear to be unique in exhibiting potent AHR activation potential. It is important to acknowledge that, in the mouse experiments presented here, we are incorporating 15% of a food in each meal, which may not represent a relevant level of dietary consumption. However, humans may consume a large amount of a vegetable in one meal (e.g., carrots), which could mediate a greater transient increase in *Cyp1a1* expression than observed in the mouse diet, where a given vegetable consumed in a meal is limited to 15% of the meal. In this study, onions and carrots at 15% were able to increase *Cyp1a1* expression, whether this is due to the presence of an AHR agonist, or another mechanism remains to be explored.

Phytochemicals are produced by plants as a defense mechanism and, in our natural environment, plants contain high levels of phytochemicals that are selectively toxic to members of the animal kingdom. The select number of plants that humans eat today have been likely bred to lower the levels of potentially toxic phytochemicals. Therefore, from an evolutionary standpoint, animals have evolved defenses against toxic plant metabolites. This would suggest that the AHR-mediated increase in the intestinal CYP1A1/1B1 levels is a protective mechanism to metabolize a wide array of chemicals in the diet prior to systemic circulation. Considering the widespread occurrence of flavonoids, many of which are AHR antagonists, it is possible that a given vegetable could have a mixture of agonists and antagonists that could yield no net change in the *Cyp1a1* levels in vivo. In the colon, the co-administration of parsley and broccoli led to the same level of AHR activation observed with broccoli alone. One would expect that apiin would be metabolized by bacteria to apigenin in the colon. Thus, a possible explanation for our results is that apigenin-mediated CYP1A1 inhibition of ICZ metabolism could increase *Cyp1a1* expression. However, the fact that apigenin is an AHR antagonist should attenuate AHR activation and thus result in, overall, no observed effect. Additionally, rapid absorption into the lymphatic compartment and/or CYP1A1-mediated metabolic clearance of ICZ within the small intestine would restrict ICZ transit to the colon and consequently restrict colonic *Cyp1a1* expression.

Many exogenously derived AHR ligands are also substrates for CYP1A1; hence, ligand metabolism limits the level of AHR activation through a negative feedback loop [39]. Based on in silico analysis, ICZ has a relatively high binding potential to CYP1A1 and docks close to the heme group; as a result, it would be highly susceptible to metabolism prior to entering the circulation from the intestinal tract [14]. We have recently published that urolithin A in mice is a CYP1A1/1B1 substrate but not an AHR antagonist. Dietary consumption of urolithin A in a 10% broccoli diet led to an increase in *Cyp1a1* in both the duodenum and the lung, while no change in the liver *Cyp1a1* was observed [14]. This study suggested that ICZ produced in situ from broccoli consumption enters the lymphatic system, providing an explanation for the *Cyp1a1* induction in the lung but not in the liver. There are likely numerous CYP1A1 substrates in foods that, when present at high concentrations, could attenuate the metabolism of a potent AHR ligand present at relatively low concentrations. One example of this is the combination of apigenin and ICZ presented in this report. However, many flavones are both AHR antagonists and CYP1A1 substrates. This complicates interpretation of the data, especially when you consider the differences in the solubility and absorption parameters for each compound. In addition, the relative potency of apigenin as a competitive AHR antagonist versus as a competitive CYP1A1 substrate will, in part, dictate which activity would be dominant compared to the same parameters for a potent AHR agonist alone, which is a CYP1A1/1B1 substrate. Apigenin is the major flavonoid in parsley, found at between 4.5 and 13% on a dry weight basis [29]. Apigenin exists in parsley largely as apiin, a diglycoside conjugate, which in the intestinal tract is deconjugated to apigenin by bacteria and by small intestinal β-glucosidases [40,41]. The dietary exposure to parsley and broccoli compared to a broccoli-only diet suggests that parsley influences the ability of a broccoli constituent to activate the AHR. The presence of apigenin, likely in a higher concentration than ICZ, would attenuate ICZ metabolism, allowing ICZ to enter the systemic circulation and activate the AHR in other tissues. Parsley alone failed to significantly activate the AHR in the intestinal tract, suggesting a lack of agonists in parsley. Importantly, we have shown that apigenin enters the systemic circulation after consumption of parsley and that the parsley diet alone is capable of modestly increasing *Cyp1a1* expression in the liver and lung. This would suggest that in these tissues, apigenin may be acting as a competitive substrate for CYP1A1, leading to the increased half-life of an endogenous or dietary AHR agonist. A particularly important aspect of CYP1A1 inhibition enhancing AHR activation is that, to see this effect, there needs to be a significant level of CYP1A1 protein present to inhibit. Indeed, the effect of parsley was greatest in tissues that are expected to have significant CYP1A1 levels, such as the liver and lung. The lung exhibits a relatively high constitutive level of *Cyp1a1* expression that is completely dependent on AHR expression [42]. This would suggest that the activation observed across various tissues may look different than in the presence of an agonist alone. For example, immune cells have limited xenobiotic metabolic capacity compared to epithelial cells and thus a CYP1A1 substrate/inhibitor would possibly have a lesser effect on the half-life of an AHR agonist.

## 5. Conclusions

Our results illustrate that testing plant extracts in cell culture models is insufficient to fully appreciate the AHR activation potential in vivo. The lack of an increase in AHR activation in vivo compared to results seen in vitro with vegetable extracts would suggest that while AHR ligands/activators are found in a variety of vegetables, other parameters, such as the bioavailability, solubility, rate of absorption and metabolism, are important considerations in vivo. However, our studies do indicate that there are vegetables that can lead to significant levels of AHR activation. In addition, the concept that CYP1A1 inhibitors/substrates in the diet can dramatically alter the level of AHR activation in tissues, especially in the lung, has been established, such as was observed here with the combination of parsley and broccoli in the diet. Nevertheless, these studies offer insights into how diet can play a key role in regulating AHR activation in a tissue-specific manner. The ability of dietary apigenin to inhibit the duodenal *Cyp1a1* levels would suggest that AHR ligand escape from the small intestinal tract can lead to an increase in *Cyp1a1* in the lung.

## Figures and Tables

**Table 1:** Computational docking analysis of apigenin in human and mouse CYP1A1.

Substrate		Computational Docking Program					
		*Autodock 4.2*				*Autodock Vina*	
		Human CYP1A1		Mouse CYP1A1		Human CYP1A1	Mouse CYP1A1
		Dissociation Constant ^a^ (K_D_) in nM	Binding Energy ^b^ (kcal/mole)	Dissociation Constant (K_D_) in nM	Binding Energy (kcal/mole)	Binding Energy ^c^ (kcal/mole) [K_D_]	Binding Energy (kcal/mole) [K_D_]
Apigenin	Max ^d^	107	−9.5	90	−9.6	−11.2 [6.3 nM]	−11.1 [7.5 nM]
	Avg ^e^	125 ± 27	−9.4 ± 0.1	110 ± 21	−9.5 ± 0.1	−10.4 ± 0.8	−10.3 ± 0.6

^a^ Dissociation binding constants (K_D_) were derived computationally using Autodock 4.2 analysis. ^b^ Substrate binding energies were derived computationally using Autodock 4.2 analysis. ^c^ Substrate binding energies were derived computationally using Autodock Vina. Because the K_D_ values are not computed manually in Autodock Vina, they were extrapolated to the Autodock 4.2 conversion scale using the following equation: y = 0.5982ln(x) − 12.304, which was derived from the Autodock 4.2 docking data presented in this table (R^2^ = 0.9994). ^d^ Max refers to the maximum affinity, or lowest-energy docking solution, found by either Autodock 4.2 or Autodock Vina. For Autodock 4.2, the solution was selected from the top 20 docking conformations (long GA runs) and from an unlimited number of solutions in Autodock Vina. ^e^ Avg refers to the average or cumulative dissociation constant K_D_ and the binding energy for all the docking poses obtained for an individual substrate:model docking combination (N < 20).

## Data Availability

The original contributions presented in this study are included in the article; further inquiries can be directed to the corresponding author. The data are not publicly available due to the ethical guidelines for animal research.

## Supplementary Materials

The following supporting information can be downloaded at https://www.mdpi.com/article/10.3390/nu17050876/s1, Figure S1: Assessment of flavonoid-mediated antagonist activity in an AHR reporter cell line; Figure S2: Flavonoids do not directly inhibit luciferase; Figure S3: Assessment of flavonoid-mediated agonist activity in a human AHR reporter cell line; Figure S4: Isorhamnetin, quercetin, luteolin, and kaempferol inhibit CEE-luciferin metabolism in the Hepa 1 microsomal CYP1A1 assay system in a dose-dependent manner; Figure S5: Dietary parsley, broccoli or a combination of the two fail to activate the AHR in the kidney; Figure S6: Apigenin docking in human and mouse CYP1B1; Figure S7: Hepa 1 microsomes are capable of hydroxylating apigenin to form luteolin. Table S1. Computational docking analysis of apigenin in human and mouse CYP1B1.

## Abbreviations

The following abbreviations are used in this manuscript:

AHR	Aryl hydrocarbon receptor
dihydroxyICZ	5H,11H-indolo[3,2-b]carbazole-2,8-diol
ICZ	Indolo[3,2-b]carbazole
TCDD	2,3,7,8-tetrachlorodibenzo-*p*-dioxin
