# Supplementary material for: Phytochemical-Mediated Ah Receptor Activity Is Dependent on Dietary Context
Source: Nutrients. 2025 Feb 28;17(5):876. doi: 10.3390/nu17050876 (PMC11901531; doi:10.3390/nu17050876)
Supplement: Supplementary file 1 [file nutrients-17-00876-s001.zip › nutrients-3475243-supplementary.pdf]

Supplementary Materials

Supplemental Table

Table S1. Computational Docking Analysis of Apigenin in human and mouse CYP1B1.

Substrate		Computational Docking Program					
		Autodock 4.2				*Autodock Vina*	
		**Human Cyp1B1**		**Mouse CYP1b1**		**Human Cyp1B1**	**Mouse CYP1b1**
		Dissociation Constant [a] (K_D) in nM	Binding Energy [b] (kcal/mole)	Dissociation Constant (K_D) in nM	Binding Energy (kcal/mole)	Binding Energy [c] (kcal/mole) [K_D]	Binding Energy (kcal/mole) [K_D]
**Apigenin**	*Max* [d]	**619**	**-8.5**	**539**	**-8.6**	**-11.8** *[2.3 nM]*	**-9.7** *[77.7 nM]*
	Avg [e]	*626 ± 8*	*-8.5 ± 0.01*	*623 ± 88*	*-8.5 ± 0.08*	*-10.9 ± 0.8*	*-8.42 ± 0.9*

a Dissociation binding constants (K_D) were derived computationally using Autodock 4.2 analysis.
b Substrate Binding Energies were derived computationally using Autodock 4.2 analysis.
c Substrate Binding Energies were derived computationally using Autodock Vina. Because K_D values are not computed manually in Autodock Vina, they were extrapolated to the Autodock 4.2 conversion scale using the following equation: $y = 0.5982\ln(x) - 12.304$, which was derived from Autodock 4.2 docking data presented in this table ($R^2 = 0.9994$).
d Max refers to the maximum affinity, or lowest-energy docking solution found by either Autodock 4.2 or Autodock Vina. For Autodock 4.2 the solution selected from the top 20 docking conformations (long GA runs) and from an unlimited number of solutions in Autodock Vina.
e Avg refers to the average or cumulative dissociation constant K_D and binding energy for all docking pose obtained for an individual substrate:model docking combination ($N < 20$).

Supplemental figures

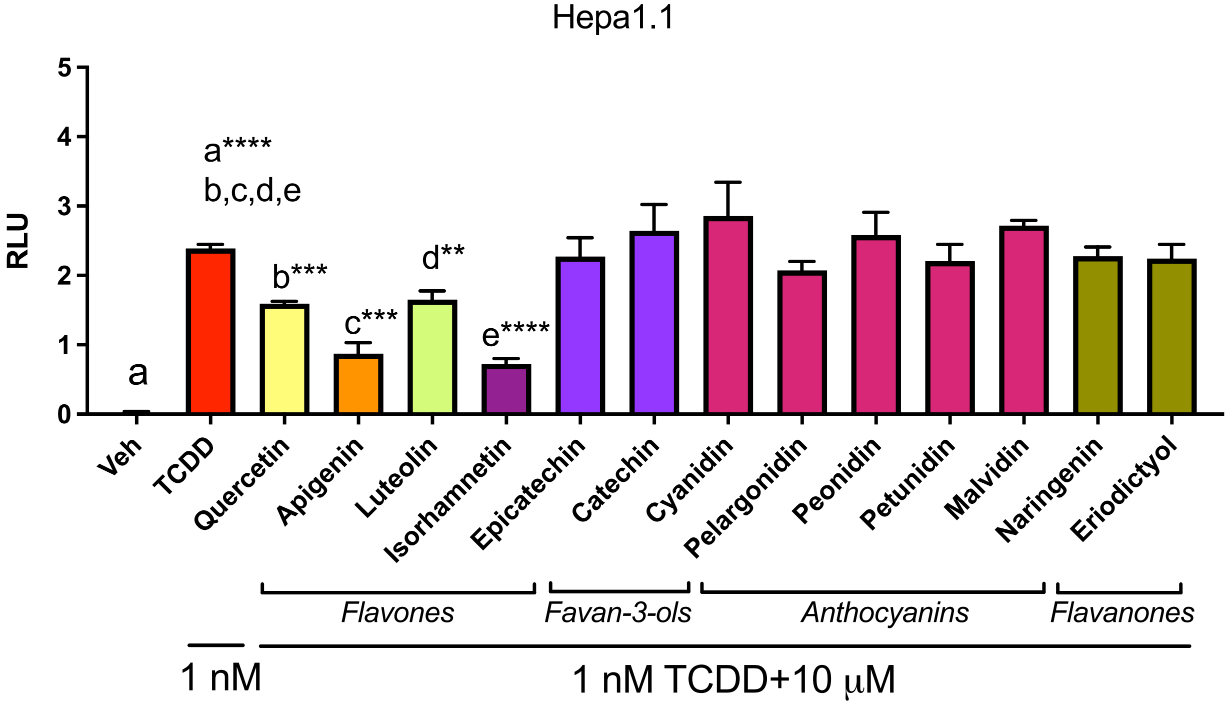

Figure S1. Assessment of flavonoid-mediated antagonist activity in an AHR reporter cell line. The ability of thirteen flavonoids to modulate TCDD-mediated AHR agonist activity was assessed in Hepa 1.1 AHR reporter cell line. Cells were treated as indicated for 4 h and luciferase activity determined. Data are presented as mean +/-S.D., significance was determined by one-way ANOVA, followed by Tukey's multiple-comparison test. ** $P < 0.01$, *** $P < 0.001$, **** $P < 0.0001$. Alphabetical characters indicate statistical comparisons between two groups.

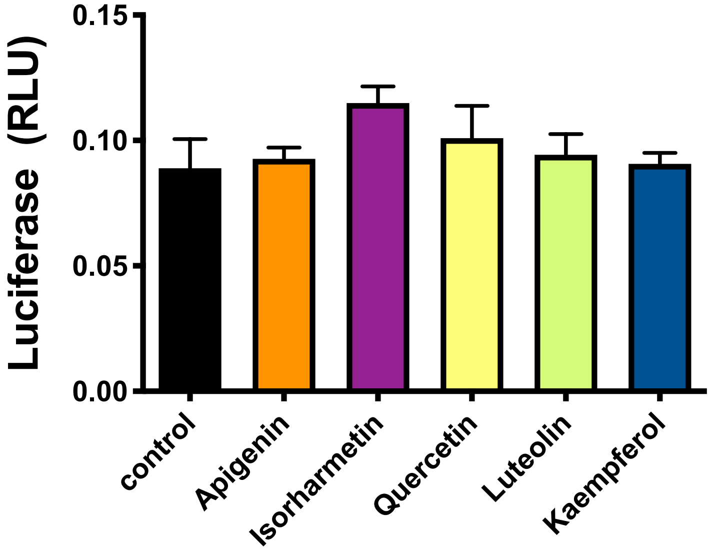

Figure S2. Flavonoids do not directly inhibit luciferase. Flavonoids at 10 μM were added to 100 μL of HepG2 40/6 lysate (1 mg/mL) that had been treated with 10 nM TCDD in cell culture overnight. Luciferase substrate was added to lysate and incubated for 30 min at room temperature. Luciferase activity was measured using a Promega luciferase assay kit as described by manufacturer.

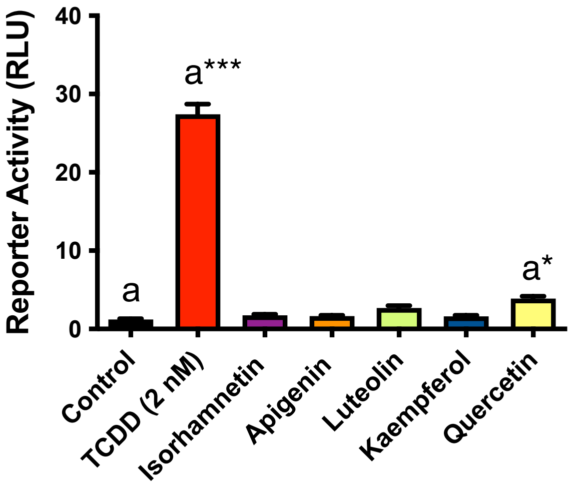

Figure S3. Assessment of flavonoid-mediated agonist and activity in a human AHR reporter cell line. The ability of five flavonoids at 10 μM to modulate AHR activity was assessed in HepG2 40/6 AHR reporter cell line. Cells were treated for 4 h with each flavonoid, compared to 2 nM TCDD as a positive control, and luciferase activity determined. Data are presented as mean +/- S.D., significance was determined by one-way ANOVA, followed by Tukey's multiple-comparison test. ** $P< 0.01$, *** $P< 0.001$. Alphabetical characters indicate statistical comparisons between two groups.

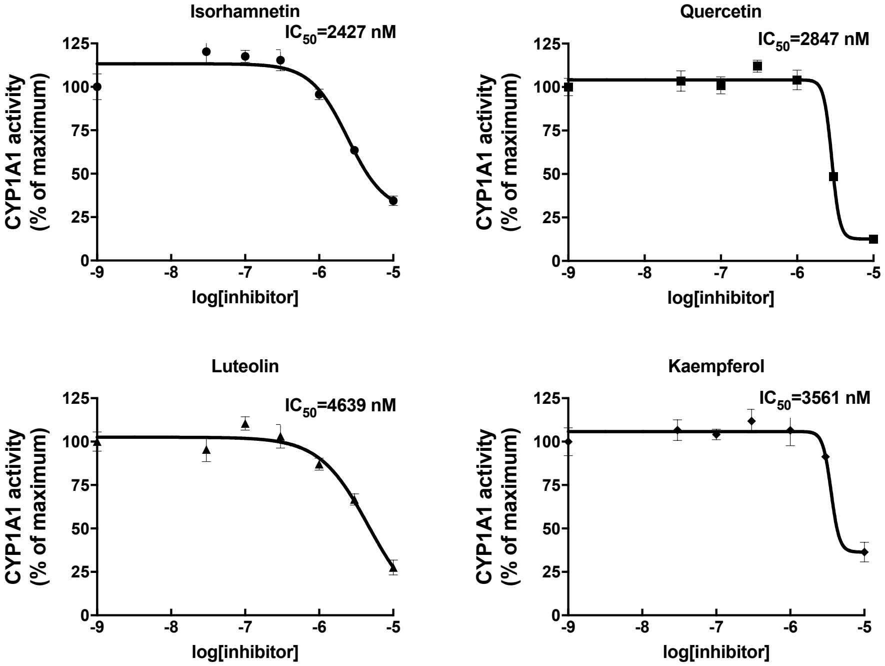

Figure S4. Isorhamnetin, quercetin, luteolin, kaempferol inhibit CEE-luciferase metabolism in Hepa 1 microsomal CYP1A1 assay system in a dose-dependent manner.

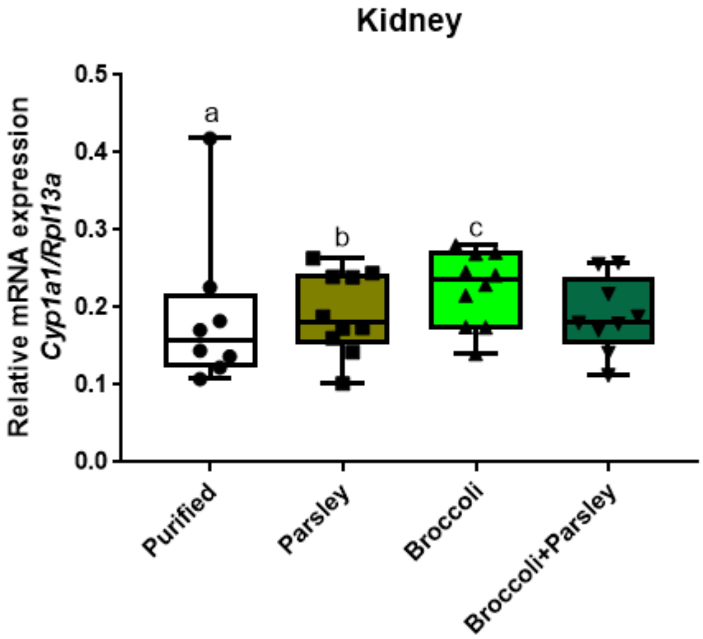

Figure S5. Dietary parsley, broccoli or combination of the two fail to activate the AHR in the kidney.

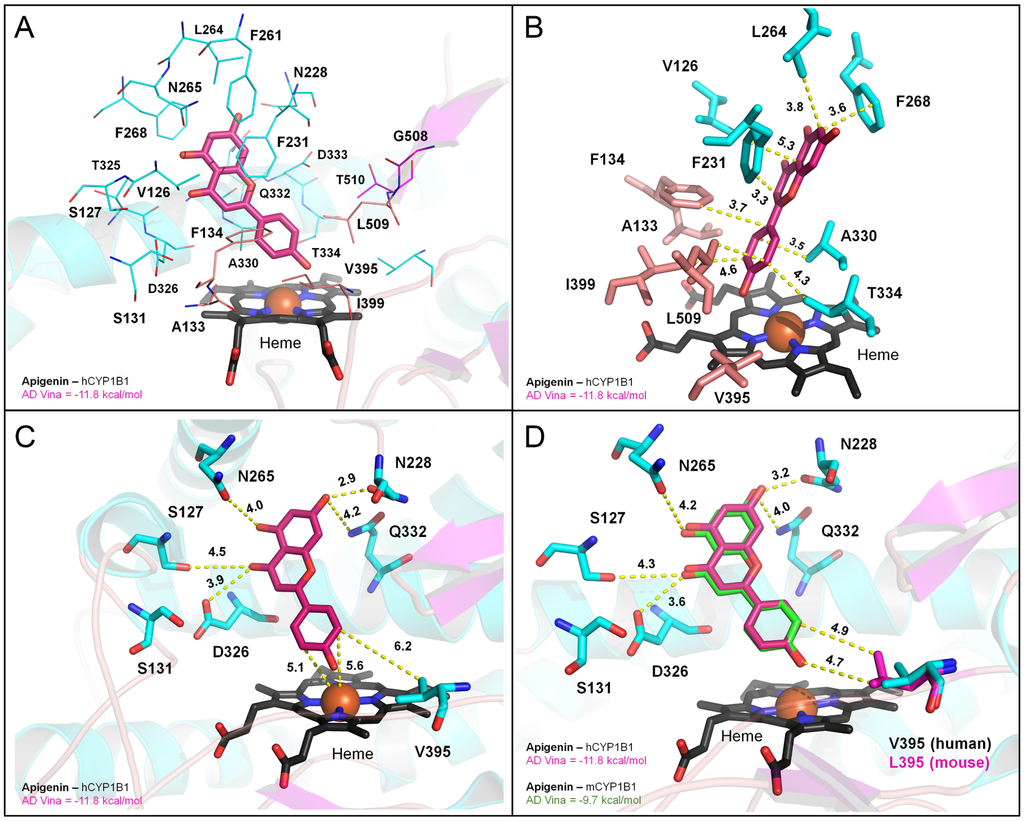

Figure S6. Apigenin Docking in Human and Mouse CYP1B1. The substrate binding properties of apigenin for human and mouse forms of CYP1B1 (hCYP1B1 and mCYP1B1) were also explored using Autodock 4.2 and Autodock Vina (see **Table 2**). **A**) Apigenin (-11.8 kcal/mol; pink stick) docks hCYP1B1 in the same common orientation observed for CYP1A1, within the horizontally compressed active site pocket formed by amino acids, V126, S127, S131, A133, F134, N228, F231, L264, N265, F268, T325, A330, Q332, D333, T334, V395, I399,G508, L509, and T510. **B**) Apigenin (-11.8 kcal/mol; purple stick) forms hydrophobic interactions with a cluster of well conserved, hydrophobic residues that flank the substrate binding pocket, including I115, F123, F224, V228, F251, L254, F258, A317, V382, L496. The distance between hydrophobic contacts is shown. **C**) Electrostatic contacts between apigenin (-11.8 kcal/mol; purple stick) and conserved, polar residues in the CYP1B1 active site are also depicted; C3 or C5 target carbons of the apigenin B-ring are shown positioned 5.1 to 5.6 Å from the heme center, nearly identical to the terminal substrate positioning observed in CYP1A1 (see **Figure 4C**). **D**) Autodock Vina predicted similar docking for apigenin in both human (-11.8 kcal/mol; pink stick) and mouse (-9.7 kcal/mol; green stick) models of CYP1B1, but there was a notable reduction in substrate binding affinity seen for apigenin in the mouse model. This reduced affinity appears to be linked to altered interactions between the substrate's B-ring and species-specific amino acid differences in the β1-4 sheet, at residue V395, which is L395 in the mouse. Increased repulsion of the C4-hydroxyl group in apigenin by L395 may explain the reduced binding affinity seen here.

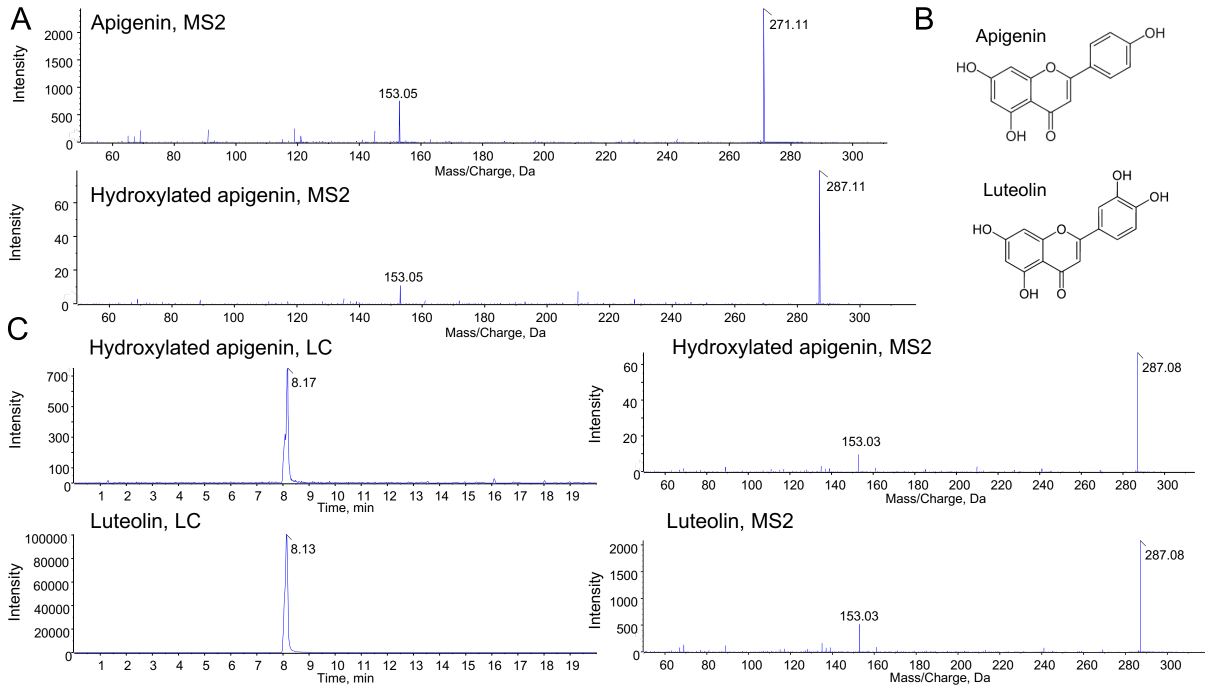

Figure S7. Hepa 1 microsomes are capable of hydroxylating apigenin to form luteolin. Hepa 1 microsomes were incubated with apigenin and the presence of luteolin was determined by LC-MS/MS analysis.
